# Deletion of pancreatic β‐cell adenosine kinase improves glucose homeostasis in young mice and ameliorates streptozotocin‐induced hyperglycaemia

**DOI:** 10.1111/jcmm.14216

**Published:** 2019-05-01

**Authors:** Makawi Ahmed Abdalhamid Osman, Yu‐Jing Sun, Rui‐Jia Li, Hui Lin, Dong‐Mei Zeng, Xin‐Yu Chen, Dongfang He, Hui‐Wei Feng, Zhao Yang, Jin Wang, Chaodong Wu, Min Cui, Jin‐Peng Sun, Yuqing Huo, Xiao Yu

**Affiliations:** ^1^ Department of Physiology and Pathophysiology Shandong University School of Basic Medical Sciences Jinan Shandong China; ^2^ Department of Physiology, Faculty of Medicine and Health Sciences University of Dongola Dongola Sudan; ^3^ Key Laboratory Experimental Teratology of the Ministry of Education, Department of Biochemistry and Molecular Biology Shandong University School of Basic Medical Sciences Jinan Shandong China; ^4^ The Second Hospital of Shangdong University Jinan Shandong China; ^5^ Department of Pharmacology Shandong University School of Basic Medical Sciences Jinan Shandong China; ^6^ Department of Nutrition and Food Science Texas A&M University College Station Texas; ^7^ Department of Biochemistry, School of Medicine Duke University Durham North Carolina; ^8^ Department of Cellular Biology and Anatomy, Vascular Biology Center Medical College of Georgia, Augusta University Augusta Georgia

**Keywords:** adenosine kinase, diabetes, insulin, replication, β cell

## Abstract

Severe reduction in the β‐cell number (collectively known as the β‐cell mass) contributes to the development of both type 1 and type 2 diabetes. Recent pharmacological studies have suggested that increased pancreatic β‐cell proliferation could be due to specific inhibition of adenosine kinase (ADK). However, genetic evidence for the function of pancreatic β‐cell ADK under physiological conditions or in a pathological context is still lacking. In this study, we crossed mice carrying LoxP‐flanked Adk gene with Ins2‐Cre mice to acquire pancreatic β ‐cell ADK deficiency (Ins2‐Cre^±^Adk^fl/fl^) mice. Our results revealed that Ins2‐Cre^+/‐^Adk^fl/fl^ mice showed improved glucose metabolism and β‐cell mass in younger mice, but showed normal activity in adult mice. Moreover, Ins2‐Cre^±^Adk^fl/fl^ mice were more resistant to streptozotocin (STZ) induced hyperglycaemia and pancreatic β‐cell damage in adult mice. In conclusion, we found that ADK negatively regulates β‐cell replication in young mice as well as under pathological conditions, such as STZ induced pancreatic β‐cell damage. Our study provided genetic evidence that specific inhibition of pancreatic β‐cell ADK has potential for anti‐diabetic therapy.

## 1 INTRODUCTION

Both type 1 and type 2 diabetes ultimately result from the failure of pancreatic β‐cell functions, including either a reduced β‐cell mass or insufficient insulin secretion in response to high blood glucose.^1^, ^2^, ^3^, ^4^, ^5^, ^6^ However, the β cells of human islets have a very short proliferation window, which normally ends after childhood.^5^, ^7^, ^8^, ^9^, ^10^ Therefore, diabetes patients normally cannot compensate for islet β‐cell loss and there is great interest in searching for pharmacological reagents to increase β‐cell proliferation under different diabetic pathological conditions.^4^, ^5^, ^11^

Recently, using the high‐throughput primary β‐cell proliferation assay, two adenosine kinase (ADK) inhibitors (5‐IT) and ABT‐702 were shown to effectively improve β‐cell replication.^11^ Additionally, a study has shown that 5‐IT might increase the β‐cell mass through non‐specific inhibition of dual‐specificity tyrosine phosphorylation‐regulated kinase‐1A (DYRK1A) and glycogen synthase kinase 3 beta (GSK3B). Furthermore, it was demonstrated that specific blockade of DYRK1A increases pancreatic β‐cell proliferation.^4^, ^5^ Although these studies demonstrated that specific inhibition of kinase in pancreatic β cells have the potential to effectively treat diabetes by inducing pancreatic β‐cell proliferation, genetic evidence is urgently needed to confirm the specific role of each kinase in β‐cell proliferation in different pathological processes and help in the precise and rational design of anti‐diabetic therapies.

As the first kinase target identified through high‐throughput screening for pancreatic β‐cell proliferation, ADK is a purine ribonucleoside kinase that converts adenosine to adenosine monophosphate. Adenosine kinase is ubiquitously expressed, and studies of its functional role are normally achieved by specific ablation of its gene in different tissues.^12^, ^13^, ^14^, ^15^, ^16^, ^17^ Therefore, in this study, we specifically deleted ADK expression in pancreatic β cells by crossing the Adk^fl/fl^ mice with Ins2‐Cre mice. The results demonstrated that ADK ablation in pancreatic β cells improves glucose tolerance and β‐cell function in mice. Moreover, loss of ADK in β cells increased the resistance of mice to streptozotocin (STZ) treatment, an animal model mostly mimicking type 1 diabetes.

## 2 RESULTS

### 2.1 Age‐dependent decrease in ADK expression in pancreatic islets

**Figure 1:**
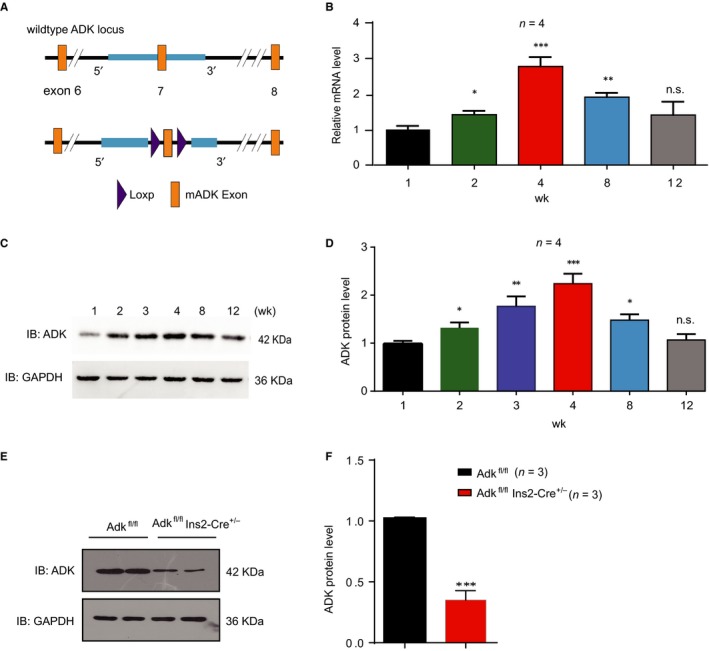
Temporal expression of adenosine kinase (ADK) in pancreatic islets and ablation of ADK in pancreatic β cells. A, Schematic representation of insulin‐producing, cell–type‐specific Adk knockout male mice (Ins2‐Cre^±^; Adk^fl/fl ^mice). The LoxP sequences were recognized by the Cre enzyme, the expression of which was driven by the insulin promoter (Ins2‐cre), thus cleaving exons 3‐7 of the Adk gene in Ins2‐cre; Adk^fl/fl ^mice and generating an Adk knockout in insulin‐expressing cells. B, The mRNA levels of ADK, as determined by quantitative reverse transcription‐PCR (qRT‐PCR), were higher in 4‐wk‐old mice than in mice of different ages (1, 2, 8 and 12‐wk‐old; *P*‐value = 0.001) n = 4. (C,D) Western blotting results and quantitative data of ADK expression showed declined expression in 1, 2, 3‐wk‐old mice but higher expression in 4‐wk‐old mice and gradually declining expression from 8 wks of age (*P*‐value = 0.003; n = 4 mice per group). Representative Western blots from at least three independent experiments are shown. (E,F) Western blots and quantitative data for ADK protein levels in islets from 4‐wk‐old mice shown from three study groups (Adk^fl/fl^, Ins2‐Cre^±^ and Ins2‐Cre^±^Adk^fl/fl^), n = 3 per group, representative Western blots from at least three independent experiments showed a significant reduction in the ADK protein expression level in Ins2‐Cre^±^Adk^fl/fl^ mice compared with control group (*P* value ≤ 0.001)*.* Note: *, ^#^<0.05; **, ^##^ *P* < 0.01; ***, ^###^ *P* < 0.001 were considered significant; *comparison of either Ins2‐Cre^±^ or Adk^fl/fl^ to Ins2‐Cre^±^Adk^fl/fl^; ^#^comparison between Ins2‐Cre^±^ and Adk^fl/fl^

Previous studies have shown that inhibition of the kinase activity of ADK leads to pancreatic β‐cell replication in isolated islets, suggesting that it is an important regulator of pancreatic β‐cell homeostasis. To characterize the temporal expression of ADK in islets, we measured the expression levels of ADK at the ages of 1, 2, 3, 4, 8, and 12 weeks. Both the mRNA and protein levels of ADK were lower in islets of 1 and 2‐week‐old mice, while higher expression was noted at the age of 4 weeks, and gradual decrease in expression was observed at the age of 8 weeks (Figure 1A‐C).

### 2.2 ADK ablation in pancreatic β cells improves glucose tolerance and β‐cell function in mice

To study the ADK function in pancreatic β cells in vivo, we crossed Adk^fl/fl^ mice with Ins2‐Cre mice to generate β‐cell specific ADK deficient Ins2‐Cre^±^ Adk^fl/fl^ mice. The Ins2‐cre^±^ Adk^fl/fl^ mice were born at expected Mendelian frequencies and had normal weights. Compared with their wild‐type (WT) littermates, the ADK protein levels in the islets of Ins2‐cre^±^ Adk^fl/fl ^mice were decreased by 65%, as examined by Western blotting (Figure 1D, E).

**Figure 2:**
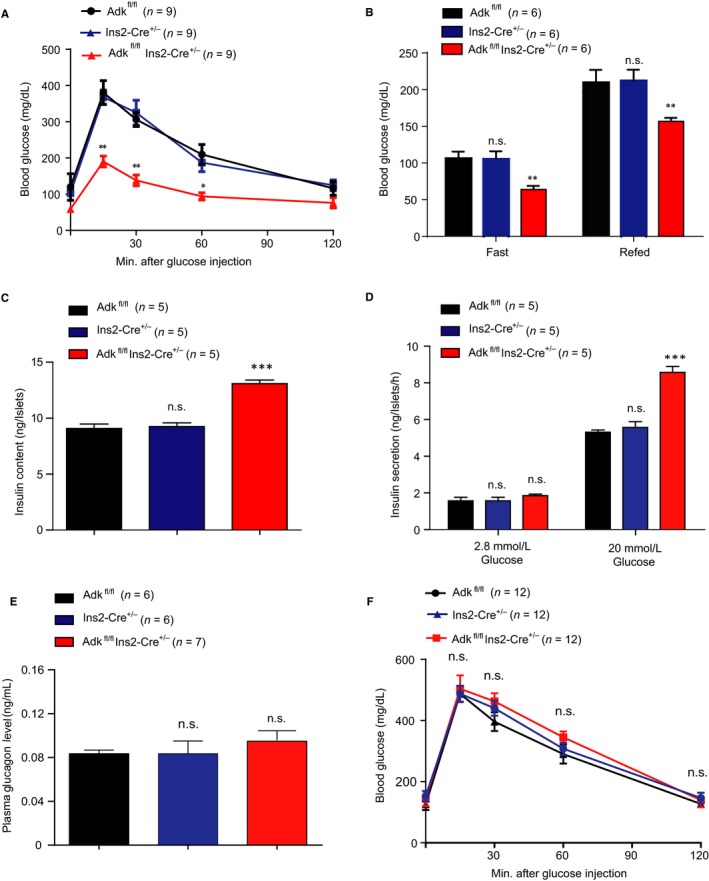
Ablation of adenosine kinase (ADK) in pancreatic β cells improves glucose metabolism. A, Glucose tolerance test results of 4‐wk‐old Adk^fl/fl^, Ins2‐Cre^+/-^ and Ins2‐Cre^±^Adk^fl/fl^ mice. Ins2‐Cre^±^Adk^fl/fl^ showed a significant reduction in the blood glucose level compared with Adk^fl/fl^ and Ins2‐Cre^+/-^ mice (*P* value ≤ 0.001); n = 9 per group. B, The fasted and fed results of 4‐wk‐old Adk^fl/fl^, Ins2‐Cre^+/-^ and Ins2‐Cre^±^Adk^fl/fl^ mice. Under the fasting condition (16 h), significant differences were shown (*P*‐value ≤ 0.001). Additionally, under the fed condition, Ins2‐Cre^±^Adk^fl/fl^ mice showed statistically lower glucose levels than Adk^fl/fl^ and Ins2‐Cre^+/-^ mice (*P*‐value ≤ 0.001); n = 6 per group). C, The insulin content of islets from 4‐wk‐old mice showed a statistically significant increase in Ins2‐Cre^±^Adk^fl/fl^ mice compared with their Adk^fl/fl^ and Ins2‐Cre^+/-^ littermates (*P*‐value ≤ 0.001); n = 5 per group. D, Insulin release from 4‐wk‐old mouse islets treated with a low‐glucose or high‐glucose dose for 1 h. Ins2‐Cre^±^ Adk^fl/fl^ showed a statistically significant increase (*P*‐value ≤ 0.001) in insulin secretion compared with the littermate control group, n = 5 per group. E, Quantification data of plasma glucagon concentration showed no significant differences among the study group; n = 6‐7 mice per group. F, Glucose tolerance test for 9‐ to 12‐wk‐old Adk^fl/fl^, Ins2‐Cre^+/-^ and Ins2‐Cre^±^Adk^fl/fl^ mice (n = 12 per group). Ins2‐Cre^±^Adk^fl/fl^ showed no significant differences compared with the control group. Note: *, ^#^<0.05; **, ^##^ *P* < 0.01; ***, ^###^ *P* < 0.001 were considered significant; *comparison of either Ins2‐Cre^±^ or Adk^fl/fl^ to Ins2‐Cre^±^Adk^fl/fl^; #, comparison between Ins2‐Cre^±^ and Adk^fl/fl^

Next, we examined the glucose metabolism of Ins2‐cre^±^Adk^fl/fl^ mice at the age of 4 weeks and compared them with their WT littermates. In the glucose tolerance test, Ins2‐cre^±^ Adk^fl/fl^ mice showed significantly lower glucose levels than their littermate control groups, Ins2‐cre^±^ and Adk^fl/fl^ at 4 weeks of age (Figure 2A). We also assessed the blood glucose levels after 16 hours of fasting and again 1 hour later after feeding. The fasted and refed blood glucose levels were lower in the Ins2‐cre^±^Adk^fl/fl ^mice than those in the control mice (Ins2‐cre^±^ and Adk^fl/fl^) at 4 weeks of age (Figure 2B). To study whether the loss of ADK in pancreatic β cells will improve the glucose metabolism in adult mice, we tested the glucose tolerance in Ins2‐cre^±^Adk^fl/fl^ mice and their WT littermates at 9‐12 weeks of age. However, these Ins2‐cre^±^Adk^fl/fl^ mice showed no significant difference in glucose metabolism compared with their WT littermates (Figure 2F). To investigate the underlying mechanisms of the glucose metabolism change in Ins2‐cre^±^ Adk^fl/fl^ mice, we isolated the islets from the Ins2‐cre^±^Adk^fl/fl^ mice and their WT littermates at 4 weeks of age. Compared with their WT littermates, the insulin contents of pancreatic islets derived from Ins2‐cre^±^Adk^fl/fl^ mice were increased (Figure 2C). Moreover, more insulin release was observed for isolated islets derived from Ins2‐cre^±^Adk^fl/fl^ mice than their WT littermates in response to 20 mmol/L glucose stimulation (Figure 2D). To figure out if ablation of Adk in β cells may affect pancreatic alpha cell homeostasis, we measured the plasma glucagon concentration from Ins2‐cre^± ^Adk^fl/fl^, Adk^fl/fl ^and Ins2‐cre^± ^mice and found no significant differences in the plasma glucagon concentration among the study groups (Figure 2E). Taken together, these results suggest that ADK deficiency in pancreatic β cells improves glucose tolerance in response to high glucose, likely due to the increased insulin content and secretion from pancreatic islets.

### 2.3 ADK ablation induces β‐cell proliferation and islet expansion

**Figure 3:**
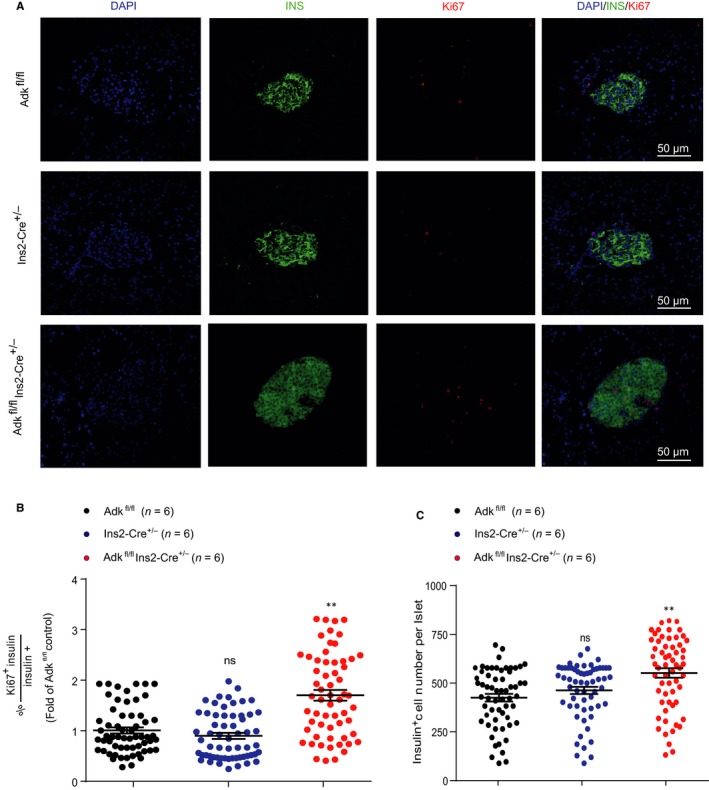
Ablation of Adk in pancreatic β cells promotes β‐cell proliferation and increased β‐cell number. Immunostaining for insulin (green) and Ki67 (red) in pancreatic sections from Adk^fl/fl^, Ins2‐Cre^+/-^ and Ins2‐Cre^±^Adk^fl/fl^ mice. Scale bar: 50 μm. n = 3 mice per group and the experiment was repeated twice; all sections were used from each mouse. B, Quantitative data showed the co‐localization of Ki67 (red) and insulin (green) in β cells. The ki67^+^ β‐cell was more abundant in Ins2‐Cre^±^Adk^fl/fl^ mice than in their littermate control groups (*P* value ≤ 0.01). C, Quantitative data of immunostaining for Ins2‐Cre^±^Adk^fl/fl^ displayed a significant increase in the β‐cell number compared with the Adk^fl/fl^ and Ins2‐Cre^+/-^ groups (*P* value ≤ 0.01). Note: *, ^#^<0.05; **, ^##^ *P* < 0.01; ***, ^###^ *P* < 0.001 were considered significant; *comparison of either Ins2‐Cre^±^ or Adk^fl/fl^ to Ins2‐Cre^±^Adk^fl/fl^; ^#^comparison between Ins2‐Cre^±^ and Adk^fl/fl^

To explore the cellular mechanisms of increased insulin content and insulin secretion of pancreatic islets isolated from Ins2‐cre^±^Adk^fl/fl^ mice, we first inspected the islet morphology and proliferation. We stained pancreatic sections with insulin and Ki67 antibodies, and found that the islets derived from Ins2‐cre^±^Adk^fl/fl^ mice showed more Ki67 staining than islets derived from their WT littermates, suggesting more proliferation of pancreatic β cells in Ins2‐cre^±^Adk^fl/fl ^mice (Figure 3A,B). Consistently, the Ins2‐cre^±^Adk^fl/fl^ mice have more pancreatic β cells in each islet statistically (Figure 3C).

**Figure 4:**
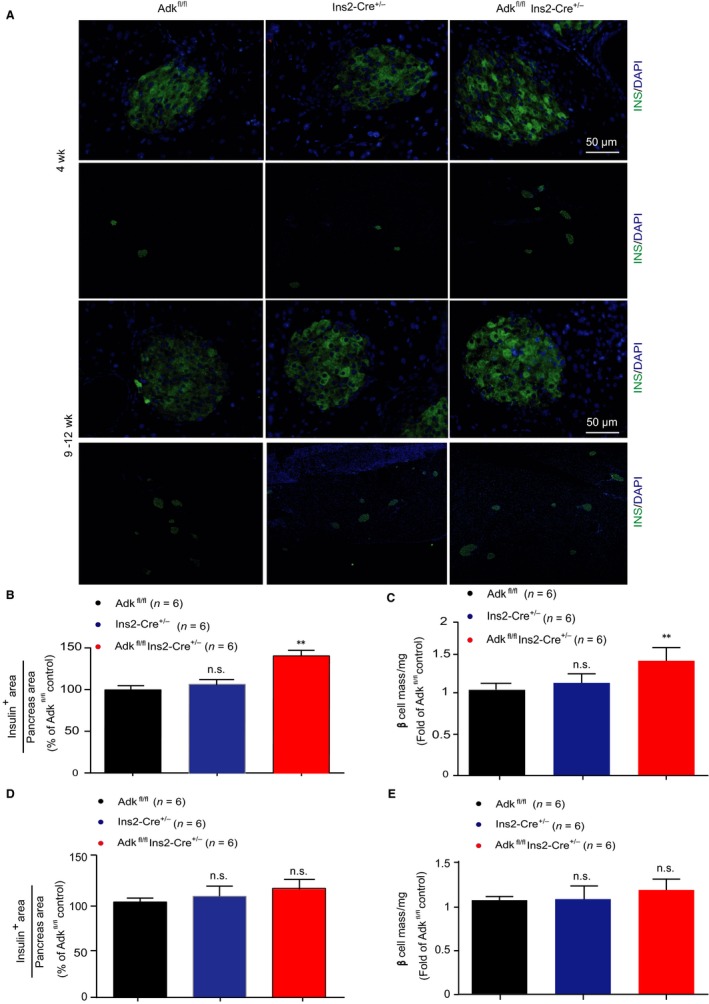
The relative β‐cell volume and β‐cell mass are increased in young Adk knockout mice. A, Immunostaining of insulin (green) and DAPI staining (blue); in pancreatic sections from Adk^fl/fl^, Ins2‐Cre^+/-^ and Ins2‐Cre^±^Adk^fl/fl^ mice (4 wks old, the upper part; 9‐12 wks old, lower part). Scale bar used: 0.5 mm and 50 μm, (n = 6 mice); all sections were used from each mouse. B, Quantitative data of the percentage of the insulin area to the total tissue area among Adk^fl/fl^, Ins2‐Cre^+/-^ and Ins2‐Cre^±^Adk^fl/fl^ young mice. C, Quantitative data for the β‐cell mass among the young study groups. (D,E) Quantitative data of the percentage of the insulin area to the total tissue area and quantitative data for the β‐cell mass among Adk^fl/fl^, Ins2‐Cre^+/-^ and Ins2‐Cre^±^Adk^fl/fl^ adult mice. No obvious differences were observed among the study groups (*P* value ≥ 0.05). Note: *, ^#^<0.05; **, ^##^ *P* < 0.01; ***, ^###^ *P* < 0.001 were considered significant; *comparison of either Ins2‐Cre^±^ or Adk^fl/fl^ to Ins2‐Cre^±^Adk^fl/fl^; ^#^comparison between Ins2‐Cre^±^ and Adk^fl/fl^

Next, we calculated the distribution of the islet β‐cell mass in knockout mice. Compared with their WT littermates, larger islets in Ins2‐cre^±^ Adk^fl/fl^ mice predominantly exist.^18^ Specifically, at 4 weeks of age, the total β‐cell mass in these Ins2‐cre^±^ Adk^fl/fl^ mice was approximately 2‐fold higher than that in their WT littermates (Figure 4A,C), consistent with the observed increased β‐cell proliferation and β‐cell number in each islet (Figure 3). We also performed islet insulin and DAPI immunostaining, targeting adult mice (9‐12 weeks of age) and found no significant differences in both β‐cell area and β‐cell mass in Ins2‐cre^±^Adk^fl/fl ^mice and their littermate (Figure 4A). Taken together, these results provided the evidence that the increased pancreatic β‐cell proliferation and β‐cell number were the cause of the increased insulin secretion identified in Ins2‐cre^±^Adk^fl/fl^ mice.

### 2.4 Islets α and δ cells numbers were comparable among the study groups

**Figure 5:**
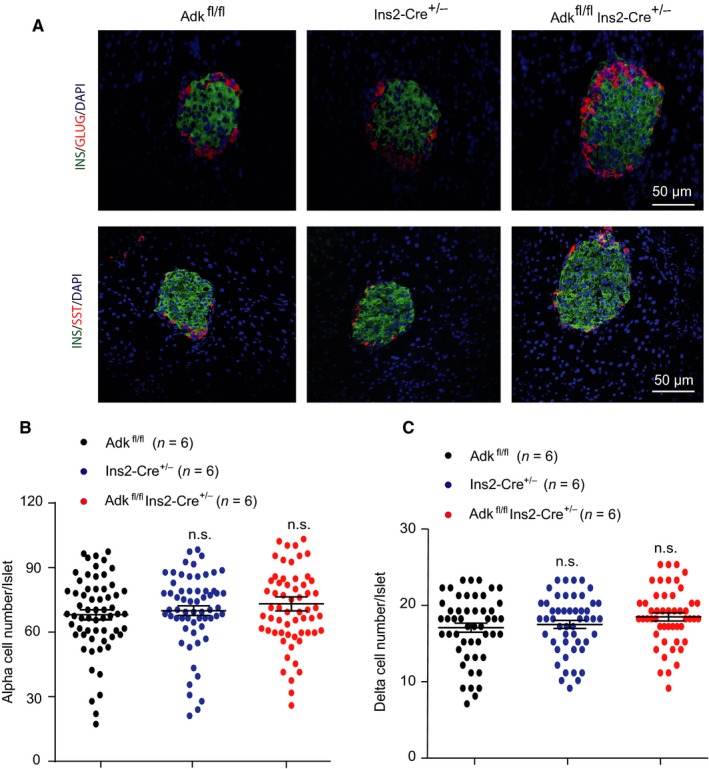
α and δ cells were comparable among the study groups. A, A representative immunostaining result for insulin (INS‐green), glucagon (GLUG‐red) and somatostatin (SST‐red) in pancreatic sections from 4‐wk‐old Adk^fl/fl^, Ins2‐Cre^+/-^ and Ins2‐Cre^±^Adk^fl/fl^ mice. Scale bar: 50 μm. (B,C) Quantification data of the islet α and δ cell number by immunostaining (n = 6 mice per group). Ins2‐Cre^±^Adk^fl/fl ^mice were comparable to their Adk^fl/fl ^and Ins2‐Cre^+/-^ littermates (*P* value ≥ 0.05)

Islets immunostaining experiments were performed, targeting 4‐week‐old (Ins2‐cre^±^Adk^fl/fl^, Ins2‐cre^±^ and Adk^fl/fl^) mice. Insulin, glucagon and somatostatin were immunostained to determine whether the increased islet size in Ins2‐cre^±^Adk^fl/fl ^mice at this age, was due to the expansion of β cells only or to the contribution of other cells (α and δ cells). Our immunostaining results demonstrated no significant changes in the number of both α and δ cells (Figure 5A‐C), confirming that the increased islet size is due to an increased β‐cell number and area.

### 2.5 Loss of ADK in a β‐cell makes the islets more resistant to STZ

**Figure 6:**
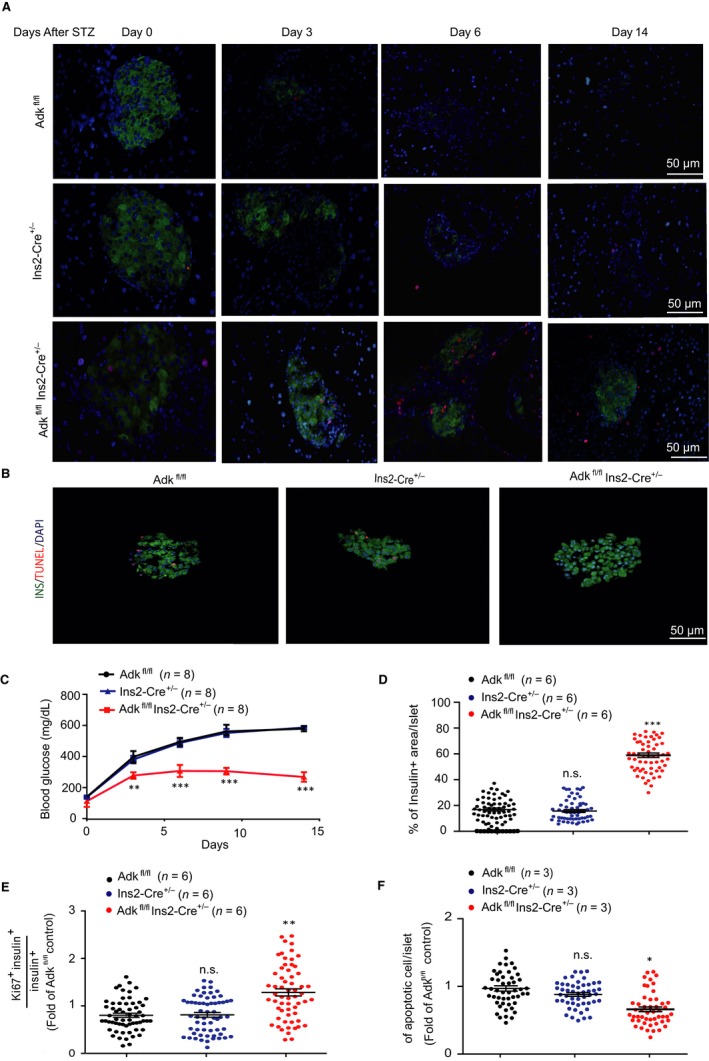
Ablation of adenosine kinase (ADK) in pancreatic β cells resists streptozotocin (STZ)‐induced hyperglycaemia through increased β‐cell proliferation. A, Immunostaining for insulin (green) and Ki67 (red) in pancreatic sections from Adk^fl/fl^, Ins2‐Cre^+/-^ and Ins2‐Cre^±^Adk^fl/fl^ mice after STZ treatment. Scale bar: 50 μm, all sections were selected from each mouse. The Ins2‐Cre^±^Adk^fl/fl^ mouse group revealed a significant increase in the number of Ki67^+ ^β cells compared with the control groups. B, Representative immunostaining for insulin (INS, green) and TUNEL (red) showing the morphology of apoptotic β cells in the islets from Adk^fl/fl^, Ins2‐Cre^+/-^ and Ins2‐Cre^±^Adk^fl/fl^ mice. C, Blood glucose levels of 9‐ to 12‐wk‐old Adk^fl/fl^, Ins2‐Cre^+/-^ and Ins2‐Cre^±^Adk^fl/fl^ mice (n = 8 per group). The blood glucose level was checked before and after STZ injection for 14 d. Ins‐Cre**^± ^**Adk^fl/fl^ mice showed significantly lower blood glucose levels than their Adk^fl/fl ^and Ins‐Cre^±^ littermates (*P* value ≤ 0.01). D, Quantitative data for the relative β‐cell area/islets, in the Ins2‐Cre^±^ and Adk^fl/fl^ groups sowed a highly significant decrease in the percentage of the β‐cell area/islets (*P* value ≤ 0.01) compared with that in the Ins2‐Cre^+/-^Adk^fl/fl^ group. E, Quantitative data for Ki67**^+^** β‐cells/islets. The Ins2‐Cre^±^Adk^fl/fl^ group showed a highly significant increase in the number of Ki67**^+^** β cells/islets compared with that in the control group, Adk^fl/fl^ and Ins‐Cre^±^, (*P* value ≤ 0.01). F, Quantitative analysis of the ratio of the TUNEL‐positive β‐cell to the islet β cells. The apoptotic β‐cell was counted as TUNEL and insulin positive cells (three mice per group). Ins2‐Cre^+/-^Adk^fl/fl^ mice displayed a significantly lower number of islet apoptotic cell than the Ins2‐Cre^± ^and Adk^fl/fl^ groups (*P* value ≤ 0.05). Asterisks indicate the level of statistical significance.*P ≤ 0.05; **P ≤ 0.01; ***P ≤ 0.001. Error bars are represented by Mean ± SD. All the data were analysed using one‐way ANOVA, or Student's *t* test. Note: *, ^#^ *P* < 0.05; **, ^##^ *P* < 0.01; ***, ^###^ *P* < 0.001 were considered significant; *, comparison of either Ins2‐Cre^±^ or Adk^fl/fl^ to Ins2‐Cre^±^Adk^fl/fl^; ^#^comparison between Ins2‐Cre^±^ and Adk^fl/fl^.

We then examined the role of ADK in pancreatic β cells under acute β‐cell loss, using a STZ‐induced type 1 diabetes model in adult mice (9‐12 weeks).^19^ Streptozotocin (100 mg/kg body weight), was injected intraperitoneally, and the blood glucose level was measured at the indicated intervals of time post‐STZ injection. Ins2‐cre^±^Adk^fl/fl^ mice were compared with their WT littermates. Intriguingly, the Ins2‐cre^±^Adk^fl/fl^ mice were more resistant to STZ treatment compared to their WT littermate (Figure 6B). The death rates on day 14 after STZ injection were 38%–43% of WT mice (Adk^fl/fl ^and Ins2‐cre^±^) and 20% of the Ins2‐cre^±^Adk^fl/fl^ mice. We also sacrificed the STZ‐treated mice from each study group at different time points (day 3, day 6, day 9 and day 14, after STZ injection) and then stained the islets with insulin and ki67 antibody. We found that there were more remaining pancreatic islet β cells of Ins2‐cre^±^Adk^fl/fl^ mice than their WT littermates, and the ki67 number was also significantly increased (Figure 6A,D,E). On the third day, approximately 60% of β cells per islets remained in the Ins2‐cre^±^Adk^fl/fl^ mice, whereas only approximately 10% β cells per islets were left in the WT mice^20^ (Figure 6A‐D). To elucidate the underlying mechanism regarding how ADK loss of function protects islet β cells from STZ‐induced damage, we performed an apoptosis assay for islets derived from adult mice (9‐12 weeks of age). Whole islets were treated with STZ (0.5 mmol/L), and apoptosis was evaluated by terminaldeoxynucleotidyl transferase‐mediated 2'‐deoxyuridine 5'‐triphosphate nick‐end labelling (TUNEL) staining. Our findings showed a significant reduction in the percentage of apoptotic islet cells in Ins2‐cre^±^Adk^fl/fl ^group compared with that in the Ins2‐cre^±^ and Adk^fl/fl^ mice group (Figure 6B,E). Taken together, our results showed that, although the deficiency of ADK in pancreatic β cells in mice has no significant effect on glucose tolerance in normal adult mice, the blood glucose level after STZ treatment is gradually improved.

## 3 DISCUSSION

There is a strong need to develop new therapeutic methods to increase the proliferation of pancreatic β cells while maintaining their normal identity and functions.^4^, ^5^, ^21^, ^22^ Recently, the screening of small compounds using a primary β‐cell replication assay or a human MYC (transcriptional regulator Myc‐like) expression system identified that inhibition of at least two kinase targets, ADK and DYRK1A, can compensate for β‐cell loss during type 1 or type 2 diabetes.^4^, ^5^, ^21^ However, kinase inhibitors sometimes have off‐target effects and the effect of long‐term disabling of this kinase is not known. Therefore, it is necessary to use a genetic model to evaluate ADK or DYRK1A function in pancreatic β cells in normal physiological contexts and diabetic related pathological models.

In this study, by crossing Adk^fl/fl^ mice with Ins2‐Cre mice, we have generated β‐cell specific ADK deficient Ins2‐Cre^±^Adk^fl/fl^ mice. These mice showed normal weight and improved glucose homeostasis by increased insulin secretion and β‐cell mass. Although whole‐body ADK knockout leads to embryonic lethality, our results suggest that constitutive ablation of ADK function in pancreatic β cells shows no detrimental effects. Similar to our work, another study using Rip‐Cre/Adk^fl/fl^ and Ins1‐Cre/ERT1^Lphi^/Adk^fl/fl^ mice also demonstrated no harmful effect by specific ablation of ADK in pancreatic β cells either constitutively or by acute induction.^23^ It is worth noting that whereas no glucose metabolism difference was found in adult mice, young‐aged (4‐week‐old) Ins2‐Cre^±^Adk^fl/fl^ mice improved glucose metabolism significantly. This difference in the observed glucose metabolism and β‐cell mass between 4 and 9‐12 weeks might be due to their age difference. Our study revealed a physiological improvement in glucose homeostasis at 4 weeks of age, which was due to the significant increase in β‐cell number and mass in Ins2‐Cre^±^Adk^fl/fl^ mice compared with their littermates. The adult Ins2‐Cre^±^Adk^fl/fl ^mice showed no significant increase in β‐cell number and mass at physiological condition, probably due to the effects of other regulatory factors in the post‐maturational β‐cell proliferation.^24^, ^25^ Therefore we assessed glucose homeostasis and β‐cell homeostasis at pathological condition through β‐cell ablation using STZ. Our results exhibited that Ins2‐Cre^±^Adk^fl/fl^ mice were more resistance to STZ‐induced hyperglycaemia compared with their littermates. This resistance to STZ‐induced hyperglycaemia was explained by significant increase of β‐cell proliferation which was accompanied by significant decrease in β‐cell apoptosis in Ins2‐Cre^±^Adk^fl/fl^ compared with their littermate control groups.

Importantly, the Rip‐Cre/Adk^fl/fl^ and Ins1‐Cre/ERT1^Lphi^/Adk^fl/fl^ mice showed significantly improved glucose homeostasis under high‐fat diet conditions,^23^ which mostly mimic type 2 diabetes. In our study, we found that the specific loss of ADK in a β‐cell causes Ins2‐Cre^±^Adk^fl/fl^ mice to be more resistant to STZ treatment, a pathological model generally for type 1 diabetes. Taken together, both recent studies and our studies using cell‐type‐specific genetic knockout models of ADK have provided strong evidence that targeted inhibition of pancreatic β‐cell ADK could be a useful strategy for the treatment of diabetes. Further studies developing a specific delivery strategy to selectively inhibit ADK in pancreatic β cells using an ADK inhibitor have important value for treating diabetes.

## 4 MATERIAL AND METHODS

### 4.1 Animal breeding and genotyping

The Cre driver Ins‐2‐Cre mice were bought from Jackson Laboratory as described previously.^26^ The Adk^fl/fl ^mice were provided by Professor Yuqing Huo at Vascular Biology Center, Department of Cellular Biology and Anatomy, Medical College of Georgia, Augusta University.^27^, ^28^ All experiments were performed with male mice. PCR‐based genotyping for DNA extraction from tails was performed using standard methods. Tissue from mice was obtained by tail clipping or from specific organs. Genomic DNA was prepared following standard procedures. PCR products were loaded in 2% agarose gel dyed with ethidium bromide for band visualization.

### 4.2 Islet isolation and Western blotting

Pancreatic islet isolation was performed as previously reported in our laboratory.^19^, ^26^, ^29^, ^30^, ^31^ Briefly, 4‐week‐old mice were killed by cervical dislocation, and the pancreata were removed and injected with 1 mL of Hanks buffer solution (136.9 mMol/L NaCl; 5.4 mMol/l KCl; 1.3 mMol/l CaCl2; 0.8 mMol/l MgSO4; 0.44 mMol/l KH2PO4; 0.34 mMol/l Na2HPO4; 5.55 mMol/l D‐glucose; 4.4 mMol/l NaHCO3, pH = 7.35‐7.45) containing 1 mg/mL of collagenase (Roche, Basel, Switzerland). The pancreas was processed and digested at 37°C for 15‐18 minutes, and then, the islets were moved to supplemented culture medium containing 1000 mg/L glucose, 10% fetal bovine serum (FBS) and 1% penicillin/streptomycin. The islets were collected manually under a stereomicroscope. For protein extraction, the islets were washed with cold PBS and lysed in cold lysis buffer (0.2 mmol/L ethylenediaminetetraacetic acid, 20 mmol/L N‐2‐hydroxyethylpiperazine‐N‐2′‐ethanesulfonic acid (HEPES), 420 mmol/L NaCl, 1.5 mmol/L MgCl_2_, 10 mmol/L Na3VO4, 10 mmol/L NaF, 25% (v/v) glycerol, protease inhibitor cocktail) for 20 minutes at 4°C. After centrifugation at 13800× g at 4°C for 30 minutes, the Bradford protein assay was used to quantify the protein concentration. Equal amounts of the islet proteins were denatured in 2× loading buffer and boiled at 100°C for 10 minutes. The proteins were resolved by loading 20 μg aliquots (10% gels) under denaturing conditions on SDS/PAGE, followed by wet transfer onto poly vinylidene fluoride (PVDF) membranes (Millipore, Billerica, MA). The membranes were then incubated with the primary antibody, anti‐ADK antibody (1:3000; Abcam, Cambridge, MA, USA) or anti‐glyceraldehyde‐3‐phosphate dehydrogenase (GAPDH) antibody (1:1000; Santa) at 4°C with gentle shaking overnight. After washing, the membranes were incubated with the secondary antibody for 2 hours at room temperature followed by chemiluminescence detection with the substrate from Pierce. The films were scanned, and the band intensities were quantified using ImageJ software (National Institutes of Health, Bethesda, MD).

### 4.3 RNA extraction and quantitative reverse transcription‐PCR

RNA from islets derived from Ins2‐Cre^±^Adk^fl/fl ^mice as well as from their littermates WT control group was extracted using TRIzol reagent (Invitrogen, Carlsbad, CA, USA) as described previously.^32^, ^33^ We performed cDNA synthesis using the qRT‐PCR Kit (FSQ‐101; Toyobo) and conducted quantitative reverse transcription‐PCR (qRT‐PCR) using the Light Cycler qPCR apparatus (Bio‐Rad) with Fast Start SYBR Green Master (Roche).

### 4.4 Islet insulin secretion assay and plasma glucagon measurement

Mouse islets were isolated from the Adk^fl/fl^, Ins2‐Cre^± ^and Ins2‐Cre^±^Adk^fl/fl^ groups (n = 5 mice, per group) and then divided into 30 islets for each sample, followed by culture in vitro overnight. Next, the insulin secretion assay was performed as previously described.^19^, ^26^, ^29^, ^30^, ^31^ Briefly, each set of islets (comprising 30) was washed and incubated in 1 mL of Krebs‐Hepes buffer supplemented with 2.8 mmol/L glucose for 60 minutes at 37°C in an atmosphere of 95% O_2_ and 5% CO_2_. The medium was then replaced with 1 mL of fresh Krebs‐Hepes medium supplemented with 20 mmol/L glucose, and the islets were incubated in the same previous mentioned condition for 60 minutes. Next, the cells were centrifuged at low speed, and the supernatants were measured for the insulin concentration using a mouse insulin ELISA kit. To measure the insulin content, the same study groups (Adk^fl/fl^,Ins2‐cre^±^ and Ins2‐Cre^±^Adk^fl/fl^) were used for each group of five mice, 30 islets per mouse and the islets were lysed with 50 µL of acid‐alcohol solution (1.5% v/v HCl conc. and 70% v/v EtOH) as described previously^34^, ^35^ using the IKAT10 Basic Ultra Turrax Homogenizer. After overnight incubation at 4°C, the supernatants were collected by 13,400 *g* centrifugation and were neutralized by 1 mol/L Tris (pH 7.5). The insulin levels were measured using the Millipore Rat/Mouse Insulin ELISA kit (EMD Millipore Corporation) according to the manufacturer's instructions. For plasma glucagon level determination, blood was collected from the study groups (4‐week‐old Adk^fl/fl^, Ins2‐Cre^± ^and Ins2‐Cre^±^Adk^fl/fl ^mice), and glucagon levels were detected by using the Rat/Mouse glucagon ELISA kit (cat. #EZGLU‐30 K, EZGLU‐30BK; Millipore).

### 4.5 Glucose tolerance test

Glucose tolerance tests were performed as previously described.^19^, ^26^, ^29^ Three groups of mice were fasted 16 hours, and then the baseline (0 points) blood glucose levels were measured in samples taken from the tail vein. Next, glucose (2 g/kg body weight) in sterile PBS was injected intraperitoneally, and blood glucose was measured at serial times of 15, 30, 60 and 120 minutes after injection for the glucose tolerance test using a Free Style Lite Glucose Meter (Roche).

### 4.6 Immunofluorescence

Immunofluorescence was performed as previously described.^32^, ^33^, ^36^, ^37^ The mice were sacrificed by cervical dislocation, and the pancreata were immediately dissected, washed in PBS and fixed in 4% formaldehyde at 4°C for at least 8 hours. Next, the samples were kept in different concentration of sucrose (4 hours in 10% sucrose, 8 hours in 20% sucrose and 12 hours in 30% sucrose). The tissue was embedded in OCT (TissueTek) and 5‐µm frozen sections were obtained by cryosection. The specimens were blocked using blocking buffer solution (1× PBS/5% normal donkey serum/0.2% Triton X‐100 and 5% bovine serum albumin) for 90‐120 minutes at 4°C and then were incubated overnight with anti‐insulin (1:100; eBioscience, San Diego, CA, USA) and ant‐Ki67 (1:400; Cell Signalling Technology), anti‐glucagon (1:250; Abcam), anti‐somatostatin (1:250; Abcam). After washing three times with PBS, the slices were incubated at 4°C for 4 hours with donkey anti‐rabbit antibody (cat. # A‐31572; Alexa Fluor 555; Invitrogen) or donkey anti‐rat antibody (cat. #ab150154; Thermo Fisher). Finally, the slices were stained with 4,6‐diamidino‐2‐phenylindole (DAPI 1:1000; Invitrogen), followed by three times washing with PBS, and the slides were subjected to fluorescence microscopy analysis.^35^, ^38^

### 4.7 Morphometric measurements

Immunostaining of insulin (green), glucagon (red), somatostatin (red) and Ki67 (red) was performed in pancreatic sections from Adk^fl/fl^, Ins2‐Cre^+/‐^ and Ins2‐Cre^±^Adk^fl/fl^ mice (Scale bar: 50 μm, n = 6 mice for each point; all sections were used from each mouse). The calculation of the ratio of the β‐cell area/exocrine area (through a convention called the relative β‐cell volume) was performed as previously described.^39^, ^40^ Slides from each case were examined using a Zeiss LSM 780 laser scanning inverted confocal microscope (Germany). The objective magnification used for the slide scanning was ×40. The analysis was performed by manual picture acquisition and cell counting. Image analysis was performed to quantify the total tissue area within this region, followed by the insulin‐positive area to generate the ratio of insulin staining to the total pancreas area using ImageJ and Image‐Pro Plus software. To calculate the β‐cell number per islet, each islet was evaluated to obtain the total islet cross‐sectional area and the cell number within this islet positive for insulin using Image J and Image‐Pro Plus software. To calculate Ki67^+^insulin^+ ^cell number, only Ki67 immunostaining co‐localization with DAPI in the insulin^+^ cell was counted (Tables 1&2).

**Table 1:** Reagents and kits used in the study

Reagent type (species)	Designation	Source	Identifiers
Antibody	ADK antibody	Abcam	ab38010
Antibody	GAPDH antibody	Santa	sc‐25778
Antibody	insulin Alexa Fluor 488	eBioscience	AB_2574468
Antibody	Ki67 antibody	Cell Signaling Technology	#9129
Antibody	Anti somatostatin	Abcam	ab30788
Antibody	Alexa Fluor 555	Invitrogen	Cat. No. A‐21424
Antibody	Glucagon antibody	Abcam	ab10988
Commercial assay	SYBR Green Master	Roche	491391400
Kit	Insulin ELISA kit	EMD Millipore	MA 01821
Kit	qRT‐PCR Kit	Toyobo	FSQ‐101

ADK, adenosine kinase; qRT‐PCR, quantitative reverse transcription‐PCR.

**Table 2:** PCR primers for genotyping

	Forward Primers (5′‐3′)	Reverse Primers (5′‐3′)
Cre	GGACATGTTCAGGGATCGCCAGGCG	GGACATGTTCAGGGATCGCCAGGCG
ADK	CCTCTATGAGTTGAGATCCTGTCTCC	ATTTATTAACTTTACATAGATTCAGACAG
Control	CTAGGCCACAGAATTGAAAGATCT	GTAGGTGGAAATTCTAGCATCATCC

ADK, adenosine kinase.

### 4.8 Quantification of islet‐cell apoptosis

For apoptosis evaluation, adult Adk^fl/fl^, Ins2‐Cre^+/‐^ and Ins2‐Cre^±^Adk^fl/fl^ mice (9‐12 weeks of age) weighing between 20 and 25 g were killed by cervical dislocation, and islets were isolated from pancreata of the study groups. Islets (n = 80) per case were treated with STZ (0.5 mmol/L; Solarbio; cat N # S8050) for 18 hours at 37^°^C, followed by washing and fixing with 4% paraformaldehyde. Then islets were impeded in OCT, and sliced (6 µm). The sections were evaluated with insulin immunostaining, and terminaldeoxynucleotidyl transferase‐mediated 2′‐deoxyuridine 5′‐triphosphate nick‐end labelling (TUNEL, Jiangsu KeyGEN Bio TECH Crop; Ltd., cat # KGA70613) was used for apoptosis examination. The apoptotic β‐cell ratio to the total islet β‐cell was calculated using Image ProPlus software.^41^

## 5 STATISTICAL ANALYSIS

All statistical analyses are represented as Mean ± SD. Statistical differences were determined by the two‐tailed, paired Student's *t* test when only two sets of data were present or using one‐way ANOVA in the case of data with more than two groups, followed by Dunnett's and Bonferroni's post hoc tests for multiple comparisons; using GraphPad Prism Software Inc (San Diego, CA).

## 6 STUDY APPROVAL

All experiments and animal care were reviewed and approved by the Animal Use Committee of Shandong University School of Medicine.

## CONFLICT OF INTEREST

No potential conflicts of interest regarding this work were reported.
